# Comment on: Collaborative research: population-based data and validation are necessary

**DOI:** 10.1093/bjs/znac208

**Published:** 2022-06-28

**Authors:** Vascular and Endovascular Research Network (VERN) Executive Committee, Graeme K Ambler, Louise Hitchman, Ruth A Benson, Panagiota Birmpili, Robert H J Blair, David C Bosanquet, Nikesh Dattani, George Dovell, Brenig L Gwilym, Katherine Hurndall, Matthew Machin, Sandip Nandhra, Sarah Onida, Joseph Shalhoub, Aminder A Singh, Athanasios Saratzis

*Dear Editor*

We read with interest the Gloves Off opinion piece by Martin Björck on research collaboratives^1^. Research collaboratives have become a pivotal part of delivering high-quality patient-centred research. Professor Björck highlights several points that we would like to respond to.

The author begins by focusing on fraudulent publications, highlighting that co-authors of Paolo Machiarini may regret adding their names to manuscripts which subsequently turned out to be fabricated. We would argue that the committee-based leadership model of research collaboratives, where decisions and tasks are attributed to a collective body of researchers rather than to a specific individual, actually protects against such issues. In a study in which research data originate from tens or hundreds of institutions and are processed by a core research group that assumes overall responsibility, it is difficult to see how any one rogue individual could significantly corrupt the meaning or interpretation of the collaborative effort.

Professor Björck then discusses the COVER (COvid-19 Vascular sERvice) study, stating that the authors of the European acute limb ischaemia (ALI) guidelines failed to find mention of ALI in one of the publications originating from that study^2^. It is unfortunate that the authors of the guideline did not contact the Vascular and Endovascular Research Network (VERN). If they had, we would have directed them to the publication that discusses patient presentations and outcomes (tier 2 of the COVER study)^3^, rather than the publication that Professor Björck cites, which discusses service reconfiguration during the pandemic (tier 1 of the COVER study). The tier 2 publication presents data regarding patients presenting with ALI, with those undergoing revascularization having an in-hospital mortality rate of approximately 20 per cent. We would also have been able to share significant as-yet unpublished raw anonymized data, including longer-term outcomes of patients in tier 2 of the study (paper in preparation). VERN has shared such data with other guideline committees in Europe when approached (for example, for the European Society for Vascular Surgery carotid guideline). As Professor Björck points out, research takes time, and we feel it unrealistic to expect data on 1-year outcomes of patients treated during the first pandemic wave to be in the public domain only 18 months after the start of the pandemic.

Trainee collaboratives exist in a plethora of fields now, delivering high-quality studies such as the large-scale BLUEBELLE and ROSSINI RCTs. The international COVER study was set up and delivered by VERN within the space of a few months. It would not have been possible to deliver this time-sensitive global effort without the help of a research collaborative of this magnitude, and once again we would like to formally thank our colleagues from over 50 countries around the world who contributed to COVER.

Before COVER, VERN had already demonstrated its utility by delivering several successful high-quality prospective projects, which have been multiply cited, including within national and international guideline documents, thus directly influencing patient care. These include the GIVE (Groin wound Infection after Vascular Exposure) study, which has resulted in the publication of several important pieces of work highlighting the persistently high incidence of groin wound infection in vascular surgery, identifying key risk factors affecting incidence, and developing a risk calculator that demonstrates significantly better performance in predicting this outcome compared with previous efforts. VERN is currently involved in five prospective studies being delivered across the National Health Service and internationally (4 open to recruitment, 1 in follow-up) on topics as diverse as diabetic foot attack, trauma with vascular injury, acute aortic syndrome, frailty, and major lower limb amputation. We look forward to sharing the results of these exciting studies in due course.

## Collaborators

Vascular and Endovascular Research Network (VERN) Executive Committee: Graeme K Ambler (University of Bristol, Bristol, UK), Louise Hitchman (University of Hull, Hull, UK), Ruth A Benson (University of Otago, Dunedin, New Zealand), Panagiota Birmpili (Royal College of Surgeons of England, London, UK), Robert H J Blair (Royal Victoria Hospital, Belfast, UK), David C Bosanquet (Aneurin Bevan University Health Board, Newport, UK), Nikesh Dattani (University Hospitals of Leicester, Leicester, UK), George Dovell (University of Bristol, Bristol, UK), Brenig L Gwilym (Aneurin Bevan University Health Board, Newport, UK), Katherine Hurndall (Barking Havering and Redbridge NHS Trust, London, UK), Matthew Machin (Imperial College, London, UK), Sandip Nandhra (Northern Vascular Centre, Freeman Hospital, Newcastle, UK), Sarah Onida (Imperial College, London, UK), Joseph Shalhoub (Imperial College, London, UK), Aminder A Singh (University of Cambridge, Cambridge, UK), Athanasios Saratzis (University of Leicester, Leicester, UK).

*Disclosure.* The authors declare no conflict of interest.
